# Why Do Humans Exercise? A Neuro‐Evolutionary Framework for Discretionary Physical Effort

**DOI:** 10.1002/evan.70038

**Published:** 2026-06-25

**Authors:** Miguel Ángel Rodríguez, Boris Cheval, Markus Gerber, Irene Crespo, Hugo Olmedillas

**Affiliations:** ^1^ Asturian Research Group in Performance, Readaptation, Training and Health (AstuRES) University of Oviedo Oviedo Spain; ^2^ Université de Rennes École normale supérieure de Rennes, VIPS2 Rennes France; ^3^ Department of Sport, Exercise and Health University of Basel Basel Switzerland; ^4^ Department of Functional Biology University of Oviedo Oviedo Spain

**Keywords:** costly signaling, evolutionary mismatch, hominin bioenergetics, human locomotion, physical activity, reward circuitry

## Abstract

Hominin evolution made physical activity obligatory for survival. Modern environments decoupled effort from ecological returns, generating an evolutionary mismatch that favors energy conservation over voluntary movement. This review distinguishes between subsistence‐based physical activity, the ancestral condition directly coupled to survival, and discretionary exercise, a voluntary behavior performed in the absence of immediate metabolic necessity. It delineates a neuro‐evolutionary framework characterizing discretionary exercise as the functional repurposing of neural systems originally shaped for subsistence, operating through evolutionarily conserved effort‐reward valuation circuitry that computes context‐dependent cost‐benefit trade‐offs. Three complementary evolutionary pathways mediate this process: homeostatic compensation, where exercise reinstates ancestral physiological maintenance signals; costly signaling, where exercise functions as an honest phenotypic display of quality; and reward recoding, where human mesocorticolimbic circuitry attributes abstract significance and reward to physical exertion. These mechanisms explain how neurobiological exaptation and cultural mediation enable sustained voluntary effort in contemporary populations despite the absence of ecological necessity.

## 1 Introduction

Two million years of hominin evolution established physical activity (PA) as an essential requirement for daily energy expenditure and survival [1, 2]. Selective pressures associated with foraging, persistence hunting, load carrying, and long‐distance travel forged a physiology capable of sustained aerobic endurance [3]. This physiology integrated specialized musculoskeletal adaptations and a high‐throughput metabolic system [4, 5, 6]. Ancestral environments tightly coupled the energetic cost of movement to ecological returns including resource acquisition, predator avoidance, and social cooperation. This ecological coupling marks a fundamental divergence between hominins and extant great apes. While chimpanzees, gorillas, and orangutans maintain relatively sedentary lifestyles and conservative energy budgets optimized for metabolic conservation [7, 8, 9], the elevated PA levels in humans represent a derived trait shaped by sustained directional selection. The transition to bipedalism and the adoption of hunting and gathering subsistence increased daily energy expenditure, which remained functionally tethered to survival‐related demands within a landscape of energy unpredictability [2]. In sum, hominins undertook PA primarily under conditions of metabolic necessity or for immediate fitness dividends [10].

A hominin organism calibrated for high levels of obligatory PA now operates in environments that require negligible movement [11]. Technological and economic transformations in industrialized societies severed physical effort from traditional ecological functions. Thus, caloric abundance, mechanized transport, and sedentary occupations diminished the requirement for sustained locomotion and generated a pronounced evolutionary mismatch [12]. This incongruity underlies both the pandemic of insufficient PA and the paradoxical emergence of discretionary exercise. Individuals voluntarily engage in planned and structured forms of PA for health, pleasure, or social status despite the absence of immediate survival demands. This pattern requires an evolutionary explanation because it involves the sustained engagement in energetically costly behavior without direct ecological necessity [10]. Ethnographic evidence exhibits that subsistence populations participate in ritual dances, long‐distance running, and competitive displays that exceed immediate energetic requirements. These behaviors provide social and reproductive benefits such as status, cohesion, and mate attraction [13]. The Tanzanian Hadza perform all‐night dances and long‐distance ritual runs, while Mexican Tarahumara forager‐horticulturalists routinely engage in ultra‐distance running [13, 14]. These examples show that energy minimization, although continuously present as a restraining force, is not the sole determinant of human movement behavior. The Hadza spend approximately 9 h per day in sedentary activity, paralleling the duration observed in industrialized populations. However, much of this time involves active postures such as squatting, which elicit significantly higher muscle activity than chair‐sitting [15]. This postural divergence underscores a critical mismatch between ancestral and modern sedentary behaviors, and may contribute to the lower cardiometabolic risk observed in subsistence populations despite comparable sedentary time [15, 16].

Analyzing this situation requires a distinction between subsistence‐based PA and discretionary exercise (Box 1). Subsistence PA represents the ancestral condition linked to survival and energy acquisition, whereas discretionary exercise constitutes a voluntary and structured behavior performed in the absence of immediate metabolic necessity [17, 19]. It encompasses multiple forms varying in intensity and duration, which influence energetic costs and physiological responses [20]. This difference may imply that common biological systems support both forms of activity under divergent motivational conditions. Spontaneous affective mechanisms such as play promote PA across mammals without requiring explicit self‐regulatory control. In contemporary environments, analogous neural systems are engaged through reward reinforcement, social incentives, and executive regulation to support discretionary exercise.

Box 1 **Operational definitions of physical activity, inactivity, sedentary behavior, and discretionary exercise.**

**Table:** 

Physical activity	Any bodily movement produced by the skeletal muscles that results in an increase in energy expenditure above basal or resting levels [17].
Physical inactivity	Insufficient level of PA to accomplish the current PA recommendations (150 to 300 min of moderate‐intensity PA, or 75 to 150 min of vigorous‐intensity PA per week) [18].
Sedentary behaviour	Any waking behaviour characterized by a reclining, sitting, or lying posture, and which requires an energy expenditure of less than 1.5 metabolic equivalents (MET) [18].
Discretionary exercise	A subcategory of PA that is planned, structured, repetitive, and purposeful, aimed at maintaining or improving physical fitness [17].

This review delineates a neuro‐evolutionary framework to examine the phylogenetic foundations of human movement, focusing on the interaction between selection for energy conservation and the mechanisms enabling sustained voluntary effort in modern environments. The analysis integrates comparative evidence, evolutionary bioenergetics, and neurobiological models of effort valuation to address the neural systems permitting energetically costly activity independent of survival imperatives. Prior work has established the physiological and biomechanical foundations of the human active phenotype [6, 21] and identified exercise as a regulatory input into somatic maintenance systems [19], yet the neurobiological mechanisms permitting sustained voluntary effort in the absence of survival demands remain undertheorized. The present framework addresses this gap by integrating evolutionary bioenergetics, effort valuation circuitry, and cross‐cultural behavioral evidence into a unified explanatory model. Finally, we advance three evolutionary hypotheses interpreting discretionary exercise as a derived behavioral outcome of systems originally shaped by subsistence demands, reward processing, and social signaling.

## 2 Evolutionary Bioenergetics and the Hominin Metabolic Niche

Human evolutionary biology explores how metabolic energy allocates among competing physiological functions. The hominin metabolic profile embodies a series of trade‐offs sculpted by variable resource availability and ecological pressures. Patterns of daily energy expenditure manifest the divergence between humans and other extant hominids. Great apes typically display low locomotor activity and limited daily travel distances, aligning with an energy‐conserving strategy adapted to stable ecological niches. In contrast, hunter‐gatherer populations in tropical environments maintain substantially higher levels of daily movement, often exceeding 10 km per day and accumulating step counts several times higher than industrialized populations [22]. Strikingly, daily PA levels in Western industrialized populations approach those recorded in great apes [23], underscoring the magnitude of the modern sedentary shift. Humans evolved elevated levels of PA and expanded energy expenditure to facilitate cooperative subsistence and extended life history [7, 8]. This change in metabolic organization fueled the energetic demands of encephalization while preserving somatic function [24]. Together, these shifts established a high‐throughput metabolic niche that set the hominin lineage on a fundamentally different energetic trajectory from all other extant primates.

Regulatory mechanisms governing total energy expenditure (TEE) respond dynamically to variation in PA [25]. The additive model proposes that increases in PA generate proportional elevations in TEE without triggering substantial compensatory adjustments [26]. The constrained model dictates that TEE remains within a relatively narrow range where increases in PA trigger metabolic reductions in other physiological processes [27]. Empirical evidence supports elements of both models, with the operative framework depending on ecological context. Studies conducted under controlled conditions of caloric abundance report additive effects of PA on TEE [8, 26, 28, 29], whereas free‐living populations, including those in Western societies, frequently show partial metabolic compensation consistent with the constrained model [28, 30, 31, 32].

Hominins operating in open habitats performed energetically demanding behaviors such as persistence hunting that required substantial energy expenditure interspersed with periods of reduced activity [21]. This pattern of intermittent exertion and recovery forged regulatory systems designed to balance energy expenditure with conservation [33], establishing inactivity itself as an adaptive strategy to reduce the risk of energy deficit in unpredictable environments [15]. Long‐standing selective pressures embody these patterns of rest and activity. The human phenotype integrates the capacity for sustained PA with specific neurobiological mechanisms limiting unnecessary metabolic expenditure [15, 34, 35]. The transition to industrialized environments altered the ancestral relationship between energy expenditure and ecological return. Subsistence contexts directly linked PA to resource acquisition and survival [36]. Modern environments, by contrast, provide consistent energy availability independent of physical effort [37]. This decoupling precipitates a pronounced mismatch between evolved regulatory systems and current ecological conditions [38]. Mechanisms once promoting ancestral energy conservation contribute to the reduced PA levels observed in sedentary populations [12]. Discretionary exercise thus operates in tension with ancestral energy‐allocation systems, requiring motivational and regulatory processes not reliably supported by evolved bioenergetic defaults, except where spontaneous or habitual mechanisms partially bridge this gap [35, 39].

## 3 The Human Active Phenotype and the Biological Imperative of PA

The divergence of the hominin lineage from sedentary great apes signifies a fundamental shift in primate metabolic strategy, requiring a reorganization of energy allocation to support an active phenotype. Great apes operate within a hypometabolic framework characterized by low locomotion levels and constrained energy throughput, whereas selection in the genus Homo favored increased daily TEE [8]. Compared to other primates, humans are exceptional long‐distance runners, a feature that emerged in the genus Homo approximately 2 Ma and is classically attributed to anatomical, neurological, metabolic and thermoregulatory adaptations such as an enlarged gluteus maximus and improved heat dissipation [6, 40, 41]. This transition was also underpinned by specific molecular shifts, most notably the ancestral loss of a key metabolic gene (CMP‐Neu5Ac hydroxylase) during the move to the open savannah. This genetic deletion appears to have fundamentally enhanced skeletal muscle capacity for oxygen use, resulting in increased capillary density and greater fatigue resistance, effectively priming the human lineage for high‐performance scavenging and persistence hunting [42]. This physiological divergence is further reflected in the human heart, which evolved to manage the volume stress of lifelong endurance PA rather than the pressure stress characteristic of the short bursts of activity seen in chimpanzees and gorillas. In modern sedentary environments, this derived physiology exhibits marked plasticity, and the heart progressively reverts toward an ancestral, chimpanzee‐like morphology [43].

The relationship between PA and encephalization highlights the complexity of evolutionary pressures shaping human physiology. Selection for endurance capacity in early Homo increased access to energy‐dense resources and elevated overall energetic throughput, relaxing metabolic constraints and facilitating subsequent encephalization [44]. This interpretation is consistent with comparative analyses across mammalian taxa showing a broad positive correlation between maximal aerobic capacity and relative brain size [45]. This relationship is not deterministic, as felids and chimpanzees exhibit relatively large brains despite limited endurance capacity, whereas migratory birds and large ungulates display high endurance with comparatively modest encephalization quotients [8, 45, 46]. High endurance in lineages with small encephalization quotients indicates that metabolic throughput alone does not drive brain expansion, but rather that endurance capacity created the energetic conditions permitting it in the hominin lineage [47, 48].

The present framework defines PA as a regulatory input into physiological maintenance systems rather than a behavioral output. The Adaptive Capacity Model proposes that repeated bouts of PA generate signals such as increased cerebral perfusion and peripheral metabolic byproducts for the organism to interpret as cues for maintenance and repair [49]. Key aspects of human physiology evolved to expect recurrent PA as a requirement for normal operating conditions. An ultimate evolutionary explanation for this architecture is provided by the Active Grandparent Hypothesis, proposed by Lieberman and colleagues, where selection for extended lifespan and continued reproductive value through kin support favored mechanisms linking PA to somatic maintenance [19]. Exercise, particularly endurance training that improves cardiorespiratory fitness, constitutes a potent neuroprotective intervention against age‐related cognitive decline. These benefits are mediated through an integrated cascade of systemic and molecular mechanisms, including the upregulation of neurotrophic factors, enhanced cerebrovascular function, and attenuation of neuroinflammation. By promoting neurogenesis and synaptic plasticity, regular PA helps preserve structural brain integrity and limits the pathological accumulation of molecular damage, thereby reducing the risk of neurodegenerative disease and supporting functional capacity across the lifespan [44, 50]. Through an evolutionary lens, these neuroprotective effects may represent an extension of the same maintenance‐on‐demand logic, whereby somatic investment in neural integrity is prioritized when PA signals the continued ecological relevance of the organism [51]. In the absence of such signals, these pathways may be downregulated, contributing to progressive declines in physiological function [52]. From this perspective, modern sedentary environments represent a deviation from the conditions under which human regulatory systems evolved rather than a neutral biological baseline.

This evolutionary tension is further complicated by individual genetic architectures shaped over millennia that now interact with environments for which they were never selected. Specific genomic loci may modulate dopaminergic signaling or the affective response to exertion [53, 54]. Heritability estimates for PA are not fixed biological constants, but context‐dependent parameters reflecting the extent to which genetic variation is expressed within a given population and environment [55]. The capacity of environmental conditions to override shared genetic predispositions is perhaps best illustrated by monozygotic twins, who can exhibit substantial differences in cardiorespiratory fitness as a consequence of divergent behavioral habits [56]. Understanding how these genetic predispositions interact with motivational and neurobiological systems requires examining the neural architecture underlying effort valuation and reward processing.

## 4 Neurobiological Regulation of Effort: From Ancestral Foraging to Modern Discretionary Exercise

Neural systems have regulated the allocation of physical effort throughout human evolution, calibrating behavior to persistent energetic constraints. These mechanisms favored effort expenditure when expected fitness benefits outweighed metabolic costs [57]. This pattern suggests that human behavior is organized around effort optimization rather than simple effort minimization. Although our species evolved to favor mechanical efficiency, this bias does not preclude engagement in highly demanding tasks. Evidence from subsistence and social contexts instead indicates that substantial effort is readily expended whenever perceived rewards justify the energetic cost [58, 59]. Ancestral humans performed energetically demanding activities including persistence hunting, long‐distance travel, and ritualized displays despite its associated metabolic costs [10, 14, 60]. In return, these actions yielded critical returns in resource acquisition, social cohesion, status, and reproductive opportunities [13].

The functional distinction between ancestral PA and discretionary exercise is not merely behavioral but neurobiological. Selection targeted the neural systems governing effort allocation alongside biomechanical and metabolic adaptations [40, 44, 61]. Such architecture operates through the hedonic regulation of behavior, wherein affective responses function as a common currency for biological decision‐making [62, 63]. Ancestral exertion was coupled with survival payoffs, but the modern absence of these pressures renders physical effort an uncompensated metabolic cost. This generates a negative hedonic valence that triggers conserved energy‐saving mechanisms and repositions discretionary exercise as a cognitively demanding exaptation rather than a reflexive drive.

Cognitive and contextual factors modulate the subjective cost of physical effort. Experimental evidence reveals that combining physical exertion with cognitively demanding tasks elevates perceived effort. Consistently, individuals with higher executive capacity exhibit reduced perceived costs under comparable conditions [64]. The coordination between motor and executive systems possibly evolved within a cognitively demanding foraging niche. Many forms of modern exercise lack the ecological and cognitive structure characterizing ancestral activities. For some individuals, exercise yields immediate benefits, such as acute affective enhancement, stress attenuation, and improved cognitive function, which may serve as potent intrinsic reinforcers [65]. In the absence of these immediate hedonic returns, the perceived metabolic cost remains uncompensated, requiring sustained prefrontal engagement to override the evolved drive for energy conservation.

### 4.1 Neural Circuits of Effort Valuation

Neural systems involved in effort valuation regulate the allocation of costly behavior under energetic constraints within an evolutionary framework. These systems compute context‐dependent cost–benefit trade‐offs to inform adaptive decision making rather than promoting effort inherently [66]. The valuation of effort is supported by interactions between salience‐related and reward‐related neural systems [67]. The salience network integrates interoceptive and task‐related information to modulate perceived effort through the anterior insula and the anterior cingulate cortex (ACC) [68]. The anterior insula encodes internal physiological states, and the ACC contributes to the regulation of effort allocation and behavioral persistence [69]. These integrated processes facilitate effort discounting where rewards demanding greater effort suffer subjective devaluation [70]. This bias may reflect selective pressure for mechanisms preventing unnecessary energy expenditure under resource uncertainty, and is countered by effort justification, where the investment of energy paradoxically increases the subjective worth of an outcome [71]. In this context, exertion is no longer appraised solely as a metabolic cost, but as a behavior endowed with motivational value, strengthening the association between physical effort and reward [72, 73].

The ACC interacts with dopaminergic structures, including the ventral striatum and nucleus accumbens, while receiving input from the ventral tegmental area [70, 74]. These circuits integrate expected rewards with effort costs to determine the allocation of goal‐directed behavior [75]. Neuroimaging studies reveal that activity in the ventral striatum decreases as effort requirements increase [76, 77]. This inverse relationship highlights the role of dopamine in motivating behavior toward outcomes justifying energetic investment [78]. Clinical evidence indicates that dopamine depletion amplifies perceived effort costs, while dopaminergic enhancement increases the willingness to exert effort [79, 80]. Mice selected for high levels of voluntary wheel running exhibit dynamic changes in brain monoamine systems alongside an increased behavioral propensity for activity [81]. These findings indicate that specialized neural circuitry modulates the reward and valuation of physical effort to underpin the active phenotype and provide a comparative physiological framework for understanding human variability in exercise motivation.

These valuation processes operate within broader decision systems evolved in foraging contexts. The neural computation of effort involves the dorsal ACC, which tracks environmental value and search costs, and ventromedial prefrontal regions, which adjudicate between specific behavioral alternatives [33, 82]. Despite the largely automatic nature of these processes, humans possess higher‐order control systems. Executive functions supported by the prefrontal cortex allow individuals to override effort‐avoidance tendencies under specific conditions. The associated metabolic costs make these control systems an unlikely default mode of behavioral regulation [27, 59]. The resulting neurobiological interaction explains the gap between intentions and behavior in modern environments [83, 84]. Understanding this gap requires consideration of how the same valuation circuitry processes motivational signals under fundamentally different ecological conditions.

This architecture might operate as a single domain‐general valuation system. The functional distinction between subsistence PA and discretionary exercise likely reflects a hierarchical shift in the motivational signals processed by the same effort‐arbitration circuitry, rather than separate neuroanatomical pathways. Recent neurophysiological evidence supports this domain‐general architecture, showing that both voluntary, endogenously‐driven choices and forced decisions rely on identical evidence‐accumulation and motor‐preparatory mechanisms [85]. During subsistence PA, the ACC–ventral striatum axis may receive high‐value signals directly coupled to primary biological rewards, including caloric return, predator avoidance, and immediate survival or reproductive outcomes. Under these conditions, the cost‐benefit computation yields a strongly positive valuation of physical effort, facilitating behavioral engagement without requiring sustained top‐down intervention. In contrast, discretionary exercise in post‐industrial environments presents the same circuitry with a fundamentally altered signal structure. Effort costs remain metabolically concrete and immediately encoded, whereas anticipated rewards are abstract, delayed, or socially constructed. This valuation process is not static but subject to recalibration through repeated behavioral experience. The brain can learn to find intrinsic pleasure in the exertion itself, shifting the behavior from a conscious choice to a habitual reflex. Once this bond is formed, environmental cues can trigger the behavior automatically, circumventing the requirement for continuous, higher‐order cognitive control.

The system performs the same valuation computation, but under conditions where ancestral reward signals are absent. This decoupling places disproportionate regulatory demand on prefrontal executive systems, which must supply top‐down revaluation to override the cost‐dominant response of the ACC–striatal axis [86, 87]. The result is a neurobiological asymmetry in which subsistence PA is sustained by bottom‐up reward signals embedded in the ecological context, whereas discretionary exercise requires the active recruitment of higher‐order cognitive systems [88, 89, 90] to assign value to effort in the absence of those signals.

This functional distinction might explain why discretionary exercise behavior is both possible and behaviorally constrained in modern populations, given that the conserved valuation circuitry remains intact and responsive but operates under motivational conditions that differ fundamentally from those shaping its evolutionary origin.

### 4.2 PA–Dependent Neuroplasticity and Recalibration of Effort Valuation

Regular engagement in PA induces neuroplastic adaptations that modify effort valuation and reward processing. Neural systems governing effort remain dynamic and responsive to repeated behavioral experience [91]. Higher levels of PA and cardiorespiratory fitness associate with structural and functional changes in the ACC, including increased grey matter volume and altered connectivity with limbic regions [92, 93, 94, 95]. Such structural modifications correlate with reduced perceived effort and improved regulation of affective responses during physical exertion [96, 97]. Repeated activity modulates dopaminergic signaling in the ventral striatum and ventromedial prefrontal cortex [98, 99]. Thus, individuals who engage regularly in exercise show increased willingness to invest effort, aligning with a recalibration of reward valuation [96]. However, direct cross‐cultural comparisons between WEIRD and non‐WEIRD populations on this neuroplastic process remain absent, a gap that limits the generalizability of these findings to the diverse ecological contexts in which human effort‐valuation systems originally evolved.

Signaling pathways linking peripheral activity to central processes support these adaptations. Brain‐derived neurotrophic factor contributes to synaptic plasticity and learning in murine models [100, 101]. Muscle‐derived signals including myokines provide a mechanism for communication between peripheral metabolic activity and brain function [102]. Endogenous opioid and endocannabinoid systems also contribute to the affective properties of exertion [61, 103, 104]. In this regard, mu‐opioid receptor signaling in the nucleus accumbens causally promotes high levels of voluntary wheel running in selectively bred rats [105].

These integrated systems increase the rewarding value of PA to reduce the subjective cost of effort over time. Such plasticity likely enhanced the ability of humans to adapt to environments requiring sustained or repeated physical effort. These neuroplastic mechanisms thus constitute a biological bridge between ancestral effort‐valuation systems and the motivational demands of discretionary exercise, providing the experiential substrate through which voluntary PA can become self‐sustaining in the absence of immediate ecological necessity.

## 5 Evolutionary Hypotheses Explaining Discretionary Exercise in Modern Humans

**Figure 1:**
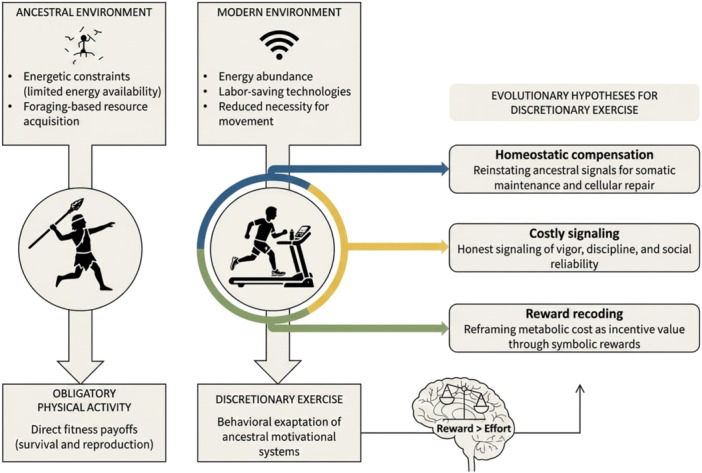
The evolutionary transition from obligatory physical activity to discretionary exercise.

The preceding analysis defines discretionary exercise as a derived behavioral outcome of multiple evolved systems interacting within novel environmental contexts rather than a direct evolutionary adaptation. Ancestral environments embedded PA within subsistence, social, and reproductive behaviors instead of performing effort for its own sake. The presence of sustained voluntary exercise in modern societies requires a mechanistic explanation regarding the redeployment of these systems outside their original adaptive context. To this end, we propose three complementary evolutionary hypotheses that differ in their current empirical standing. The homeostatic compensation hypothesis draws on the most direct evidence, combining cross‐population energetic data, molecular signaling studies, and life history theory. The costly signaling hypothesis is grounded in robust cross‐cultural behavioral and anthropological evidence, though its neurobiological substrates await direct empirical testing. The reward recoding hypothesis integrates prefrontal neuroscience and intrinsic motivation research, but remains the least directly tested in exercise‐specific contexts across ecologically diverse populations. Together, they are best understood not as competing accounts but as complementary pathways that may operate simultaneously and interact in ways that future research should seek to disentangle (Figure 1).

### 5.1 Homeostatic Compensation Hypothesis

Humans, like other animals, evolved to be physically active only when the behavior is either necessary (survival, energy balance and somatic maintenance) or rewarding [10]. Insufficient PA in industrialized societies constitutes an evolutionary mismatch that fails to trigger repair and maintenance mechanisms, thereby accelerating aging and increasing disease risk [26]. Empirical data across populations illustrate the magnitude of this transition, as evidenced by Rwandan subsistence farmers, who sustain some of the highest recorded levels of PA, with a mass‐corrected Activity Metabolic Quotient 2.6 times higher than that of urban office workers [106]. These findings confirm that humans readily perform energetically costly activity when survival necessitates the expenditure or when the behavior yields immediate social or reproductive returns [10]. In contemporary environments, the lack of these demands precipitate a mismatch between evolved biological requirements and actual behaviors. Discretionary exercise therefore constitutes a compensatory behavior reinstating the metabolic and physiological stimuli historically provided by subsistence PA. This compensatory logic is further illuminated by a life history perspective. The Active Grandparent Hypothesis proposes that selection favored mechanisms linking PA to somatic maintenance specifically during post reproductive years, when continued physical capacity enhanced inclusive fitness through kin provisioning [19]. From this perspective, the age‐related increase in health‐motivated discretionary exercise observed in industrialized populations may reflect the reactivation of maintenance pathways that obligatory subsistence PA once engaged automatically. The absence of ecological necessity does not erase the evolved architecture underlying these mechanisms, but rather removes the proximate cues that ancestrally activated them.

This hypothesis predicts that discretionary exercise will be more prevalent in populations with low obligatory PA, whereas subsistence populations will exhibit minimal structured exercise despite high total energy expenditure. Furthermore, it posits that the molecular machinery underlying somatic maintenance will reflect this compensatory logic. Repair‐related pathways such as brain‐derived neurotrophic factor and myokine signaling will be preferentially upregulated during bouts of voluntary exercise in sedentary environments, where these essential maintenance signals are otherwise chronically understimulated.

### 5.2 Costly Signaling Hypothesis

Discretionary exercise functions as a form of costly signaling through which individuals engage in energetically expensive activity to display desirable traits including physical formidability, dominance, and reproductive quality [107]. Costly signaling theory posits that such behaviors remain evolutionarily stable because high energetic costs ensure honesty. This metabolic investment makes signals difficult to fake and establishes them as reliable indicators of underlying phenotypic quality [108]. In non‐industrial societies, the physical demands of hunting and defense serve as fundamental drivers of both intra‐ and intersexual selection [109, 110]. In contemporary industrialized societies, physical fitness persists as a primary indicator of physiological resilience, long‐term health, and perceived social value [111]. In contrast, physical inactivity and sedentary behavior are frequently associated with stigma and shame [112], as they might be perceived as signals of low phenotypic quality or a lack of self‐regulatory discipline.

Theoretical models define sport as a culturally elaborated mechanism for status display and inter‐sexual assessment [113], with sporting prowess correlating positively with attractiveness and reproductive indicators in industrialized populations [114, 115]. Experimental evidence shows that individuals perform cardiovascular exercise with greater intensity and cover greater distances in the presence of potential mates. This behavioral shift occurs predominantly when individuals remain single or motivated to compete for mating opportunities [116]. Ethnographic evidence from hunter‐gatherer societies supports these patterns. Ritual runs, dances, and competitive feats remain embedded within social and reproductive contexts despite substantial energetic costs [13]. Cross‐cultural data indicate that men frequently engage in exercise to enhance muscularity and perceived dominance, while women often engage in exercise targeting body‐composition and endurance traits associated with health and vitality, patterns that evolutionary frameworks have interpreted as signals of phenotypic quality across both sexes [117, 118, 119]. These evolutionarily grounded patterns coexist with motivations rooted in health, competence, and psychological well‐being that operate independently of reproductive signaling [120].

Together, these findings suggest that discretionary exercise represents the exaptation of ancestral signaling systems originally shaped in subsistence and social contexts. Costly physical performance in these environments reliably conveyed information about individual quality. Competitive sports, endurance events, and structured training constitute culturally amplified expressions of this mechanism in modern environments. Contemporary social norms associating physical effort with discipline, virtue, and self‐control enhance the signaling value of exercise, increasing motivational salience through social evaluation and identity processes [121].

This hypothesis predicts that exercise behavior will increase under conditions of social visibility and audience presence. Furthermore, it posits that individuals will preferentially engage in status‐enhancing forms of exercise, such as competitive sports, where the display of vigor and discipline is most effectively communicated to others.

### 5.3 Reward Recoding Hypothesis

Reward systems evolved to reinforce behaviors that enhanced fitness, including food acquisition, cooperation, and reproduction. Discretionary exercise recruits these systems in modern environments when physical effort becomes associated with intrinsic rewards related to the subjective experience of meaningfulness [122] or extrinsic rewards [123]. Humans exhibit an enhanced capacity to assign value to abstract and delayed outcomes through expanded prefrontal systems. These systems permit individuals to pursue exercise for culturally constructed goals such as health, identity, and achievement even in the absence of direct survival benefits. Humans may exhibit enhanced prefrontal integration with conserved reward systems that differentiates the species from great apes. This derived circuitry may support the activation of mesocorticolimbic and thalamic pathways during goal achievement and socially valued behaviors, including athletic accomplishment, and enables the assignment of pleasure and motivational value to abstract and socially meaningful activities in the absence of instantaneous fitness returns [124, 125].

Such neural specializations may allow the subjective experience of discretionary exercise as inherently rewarding through feelings of mastery, identity, flow, or communal significance. This process substitutes for the direct fitness payoffs that once motivated ancestral PA. Repeated engagement recalibrates physical effort from a perceived cost to a source of personal significance and self‐worth. External rewards or social status may facilitate initial engagement, but sustained voluntary exercise typically requires that the activity itself be experienced as inherently meaningful.

This hypothesis predicts that initial exercise engagement may rely on external rewards, but long‐term adherence depends on satisfying basic psychological needs through intrinsic rewards and personal meaningfulness, such as enjoyment, flow, or the conviction that the effort matters. Furthermore, reward‐related regions like the ventral striatum and nucleus accumbens will show increased activation to exercise cues specifically when the activity is perceived as meaningful. Consequently, interventions enhancing the intrinsic value of exercise will yield superior long‐term maintenance compared to those focused exclusively on extrinsic rewards or health outcomes.

### 5.4 Integrative Perspective

For most of human history, natural environments shaped our biology, but the rapid pace of industrialization has now created a mismatch that outpaces our adaptive capacity [111]. We have proposed three hypotheses suggesting that discretionary exercise is not a direct evolutionary adaptation but a derived behavioral outcome rooted in the distinctive bioenergetic strategy of the hominin lineage. Humans evolved a high‐throughput metabolic engine capable of sustaining elevated TEE while retaining a strong ancestral bias toward energy conservation whenever PA is not directly linked to survival or reproductive payoffs. This dual legacy of additive metabolic capacity and constrained regulatory mechanisms provides the mechanistic foundation for all three hypotheses, each capturing a distinct pathway through which evolved systems are repurposed in post‐industrial environments.

In sum, PA evolved as a means to support survival and reproduction rather than as an end in itself. In post‐industrial environments, discretionary exercise represents a culturally scaffolded behavior that recruits and repurposes the neural and physiological systems originally shaped to regulate effort under conditions of ecological necessity and uncertainty. Homeostatic compensation drives the deliberate activation of ancestral somatic repair signals that remain dormant in modern sedentary environments. Costly signaling suggests that social and reproductive payoffs can outweigh metabolic costs. Reward recoding illustrates how intrinsic and culturally constructed valuations, shaped by prefrontal modulation and experience‐dependent plasticity, can recalibrate effort perception to permit sustained voluntary exertion without immediate adaptive incentives. The three hypotheses proposed here are not mutually exclusive, but convergent explanations for a single phenomenon, namely the capacity of an evolutionarily active organism to sustain voluntary physical effort under conditions in which ancestral survival demands no longer require it.

## 6 Conclusion

This review presents a neuro‐evolutionary framework for understanding the transition from subsistence PA to discretionary exercise. Throughout hominin evolution, PA served as an instrumental behavior strictly coupled to survival success. Technological and economic transformations in post‐industrial societies severed this ancestral link between physical effort and ecological return, generating a profound evolutionary mismatch that current biology was not shaped to resolve. Under these novel conditions, discretionary exercise emerges as a culturally mediated behavior that recruits the same neural and physiological systems originally shaped to regulate obligatory movement. The framework proposed here is primarily theoretical and invites systematic empirical validation across ecologically diverse populations. Future research would benefit from cross‐cultural studies examining the neurobiological correlates of effort valuation in both subsistence and industrialized contexts, and from longitudinal designs capable of tracking the recalibration of reward systems with exercise experience.

Sustained discretionary exercise in contemporary societies constitutes a derived behavioral phenomenon that depends on the unique human capacity to assign abstract significance and intrinsic reward to energetically costly actions. Through this capacity, the three mechanisms outlined in this review, namely homeostatic compensation, costly signaling, and reward recoding, allow humans to maintain voluntary physical effort despite the absence of immediate ecological necessity. In doing so, discretionary exercise illustrates how neurobiological exaptation and cultural mediation have enabled the persistence of the human active phenotype in a world that no longer demands it.

## Funding

The authors have nothing to report.

## Conflicts of Interest

The authors declare no conflicts of interest.

## Declarations of AI Use

Artificial intelligence–based tools were used solely to assist with language editing, clarity, and stylistic refinement of the manuscript. No AI system was used to generate original scientific content, data, analyses, interpretations, or conclusions. The authors retain full responsibility for the content, accuracy, and integrity of the work.

## Statements Relating to Ethics and Integrity Policies

This article is a theoretical and narrative review based exclusively on previously published literature. It does not involve the collection of new data, experiments with human or animal participants, or the use of identifiable personal information. As such, no ethical approval or informed consent was required. The authors declare that the work complies with the ethical and integrity policies of the journal.

## Data Availability

Data sharing not applicable to this article as no datasets were generated or analysed during the current study.
